# PEDOT:PSS-Based Conductive Textiles and Their Applications

**DOI:** 10.3390/s20071881

**Published:** 2020-03-28

**Authors:** Granch Berhe Tseghai, Desalegn Alemu Mengistie, Benny Malengier, Kinde Anlay Fante, Lieva Van Langenhove

**Affiliations:** 1 Department of Materials, Textiles and Chemical Engineering, Ghent University, 9000 Gent, Belgium; Benny.Malengier@UGent.be (B.M.); Lieva.VanLangenhove@UGent.be (L.V.L.); 2 Ethiopian Institute of Textile and Fashion Technology, Bahir Dar University, 6000 Bahir Dar, Ethiopia; dmengist@calpoly.edu; 3 Jimma Institute of Technology, Jimma University, Jimma, Ethiopia; kinde.anlay@ju.edu.et; 4 Materials Engineering Department, California Polytechnic State University, San Luis Obispo, CA 93407, USA

**Keywords:** PEDOT:PSS, wearable electronics, e-textile, conductive textile

## Abstract

The conductive polymer complex poly (3,4-ethylene dioxythiophene):polystyrene sulfonate (PEDOT:PSS) is the most explored conductive polymer for conductive textiles applications. Since PEDOT:PSS is readily available in water dispersion form, it is convenient for roll-to-roll processing which is compatible with the current textile processing applications. In this work, we have made a comprehensive review on the PEDOT:PSS-based conductive textiles, methods of application onto textiles and their applications. The conductivity of PEDOT:PSS can be enhanced by several orders of magnitude using processing agents. However, neat PEDOT:PSS lacks flexibility and strechability for wearable electronics applications. One way to improve the mechanical flexibility of conductive polymers is making a composite with commodity polymers such as polyurethane which have high flexibility and stretchability. The conductive polymer composites also increase attachment of the conductive polymer to the textile, thereby increasing durability to washing and mechanical actions. Pure PEDOT:PSS conductive fibers have been produced by solution spinning or electrospinning methods. Application of PEDOT:PSS can be carried out by polymerization of the monomer on the fabric, coating/dyeing and printing methods. PEDOT:PSS-based conductive textiles have been used for the development of sensors, actuators, antenna, interconnections, energy harvesting, and storage devices. In this review, the application methods of PEDOT:SS-based conductive polymers in/on to a textile substrate structure and their application thereof are discussed.

## 1. Introduction

With the emergence of new fibers, fabrics and innovative processing technologies, the growth of the textile market has increased in recent years and has been instrumental in bringing about significant technological advances. Starting with groundbreaking research on how to integrate conductive lines and circuits into textiles in the late 1990s, rigorous researches resulted in sensor additions, actuators, user interfaces, and complicated textile circuits that could provide extra functionality to make smart textiles. Smart textiles can be defined as textiles capable of sensing and responding to changes in their environment by external factors. In response to stimuli, they are able to show significant changes in their mechanical and/or chemical properties (such as shape, color, and stiffness), or in their thermal, optical, or electromagnetic properties. Examples include fabrics that change their color with changes in temperature and fabrics that regulate garments’ surface temperature to achieve physiological comfort. Smart materials can be incorporated into the textile structure by different technologies such as embroidering [1], non-woven textile [2], knitting [3], weaving [4], braiding [5], yarn spinning [6], fiber spinning [7], polymerizing [8], coating [9], plating [10] and printing [11].

Societal needs such as new functionality, comfort, and aesthetic values from daily use to critical health-related applications are the driving forces for the development of smart textile materials. The recent developments in the fields of textiles, electronics, information technology, advanced materials and polymers are paving the way for the development of smart textile materials and their application [12,13,14,15,16,17,18,19]. The fact that textiles are an interface between the wearer and the surrounding with large and permanent surface contact make them ideally suitable for large scale and long-term health monitoring. In addition, textile is easily accessible anywhere and has versatile applications from dressing to household products and coverings. 

The primary step in smart textiles is making conductive textiles. From a textile perspective, it is suggested that the overall objective of smart textiles would be to convert all related components, such as sensors, actuators, transmission lines, etc., into 100% textile materials [20]. However, conventional textiles are intrinsically non-conductive so they need to be converted to become conductive in some way. The earlier method of making textiles conductive was inserting thin metal fibers/yarns in the textile/garment which primarily was developed for antistatic treatment. A later development and more convenient way to make conductive textiles is to treat them with conductive inks at the polymer (man-made fibers), fiber, yarn, fabric or readymade garment stage. Conductive textiles can be classified into bulk and surface conductive textiles [21]. Bulk conductive textiles include intrinsically conductive polymer textiles, textiles twisted/embedded with metallic filaments and textiles filled with conductive additives such as carbon blacks (CBs), carbon nano-tubes (CNTs), or conductive polymers. Surface conductive textiles are the textiles coated with conductive layers. The conductive coatings may consist of metals, conductive polymers, or other conductive materials such as CNTs or CBs. From a technological point of view, the challenge is to develop a method suitable for the current textile processing, with high conductivity, durability and maintaining the desired textile properties such as flexibility. Coating with metal nanoparticles/nanowires could give high conductivity; however, it may come at the expense of flexibility and lack of durability. The focus will therefore be on enhancing durability, textile character, and conductivity. The use of electrically conductive polymeric materials have recently attracted considerable interest from academic and industrial researchers to explore their potential in sensors [22], biomedical [23], wireless communication patch antenna [24], energy harvesting [25] and energy storage [26] applications. Conductive polymers are light weight and flexible and can be applied on the textile without affecting its flexibility. Solution-based conductive polymers are especially convenient for the roll-to-roll processing which can easily be integrated with the current textile processing technologies like dyeing and printing. There are several reports [27,28,29] on the use of conductive polymers for conductive textile for different applications. Recently reported conductivities of over 6000 S/cm [30] are signaling their practical potential use in the smart textiles applications.

For this review, we made a comprehensive electronic document search according to the preferred reporting items for Systematic Reviews and Meta-Analyses (PRISMA) guidelines from the web of science database and the Google search engine. ’Conductive + textiles’, ’textile + sensor’, ’textile + antenna’, ‘textile + energy harvesting’, ‘textile + energy storage’, ’textile + interconnections’, ‘conductive + polymers’, ‘conductive + polymer + composites’, ‘e-textile’, ’PEDOT’, ‘PEDOT:PSS’, ‘EDOT’, ‘poly(3,4-ethylenedioxythiophene)’, ’poly(3,4-ethylene dioxythiophene):polystyrene sulfonate’, have all been used as primal keywords for the search.

## 2. Conductive Polymers

**Figure 1:**
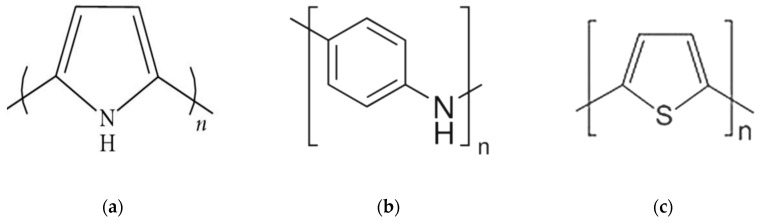
Chemical structure of the most common conductive polymers: (**a**) Polypyrrole; (**b**) Polyaniline; (**c**) Polythiophene.

Traditional commodity polymers are intrinsically insulators. The discovery of conductive polymers started with the path breaking discovery that halogen doped polyacetylene (-CH=CH-)_n_ show high electrical conductivity, which led to the 2000 Nobel Prize in Chemistry award [31]. Since then, there have been several fundamental studies and applications of conductive polymers. The carbon atom in saturated polymers, such as polyethylene, form four covalent *σ*-bonds (saturated *sp^3^*-carbon). Whereas the carbon atom in conjugated polymers has *sp^2^p_z_* (π) orbitals which form three σ-bonds and the remaining *p_z_* orbitals engage in the π system. The common feature in conductive polymers is conjugation, i.e., the alternation of single and double bonds, and hence the synthesis of π-conjugated chains is central to the science and technology of conductive polymers. The charge carriers are delocalized in conjugated systems and provide the “highway” for charge mobility along the backbone of the polymer chain. The conductivities of conjugated polymers can be enhanced by doping, which is basically either reduction or oxidation [32]. The conductivity of doped polyacetylene can reach 10^5^ S/cm which is comparable to that of copper [33]. However, polyacetylene is difficult to synthesize and is unstable in air which prevented its commercialization. The most important conductive polymer candidates currently are polypyrrole (PPy), polyaniline (PANI), and polythiophenes (PTh) whose chemical structures are shown in Figure 1. Poly(3,4-ethylenedioxythiophene) (PEDOT), which is the main topic of this article, is the most studied and successful PTh derivative polymer due to its higher electrical conductivity and chemical stability which make it suitable in the development of smart textiles [34]. In contrast to PPy and PANI, the exploration on PEDOT is comparatively recent.

Conductive polymers exhibit novel properties such as solution processability, high elasticity, toughness, and low-temperature synthetic routes. Some examples conductive polymers and their properties are presented in Table 1. Due to these interesting properties, conductive polymers are used for several applications such as photovoltaic devices [35], organic light-emitting diodes [36], organic field-effect transistors [37], sensors [38], antennas [39], conductive textiles [40], supercapacitors [41] and many more. 

## 3. PEDOT

**Figure 2:**
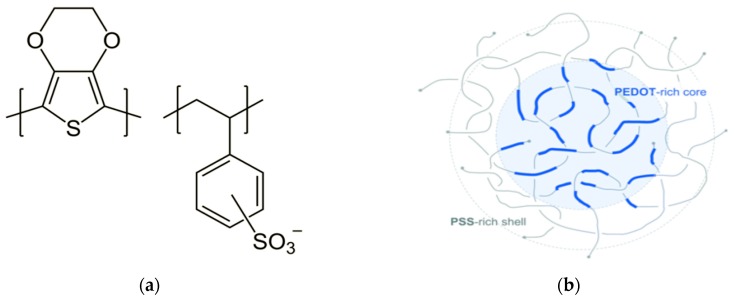
(**a**) Chemical structure of PEDOT:PSS; (**b**) core-shell structure of PEDOT:PSS, adapted from [52].

Among conductive polymers, PEDOT is the most extensively explored, successful and widely used for many applications due to its high conductivity, its stability in air up to high temperatures and resistance to humidity including moist air, and because it is also processable in water. PEDOT can be polymerized from 3,4-ethylenedioxythiophene (EDOT) chemically or electrochemically. However, PEDOT synthesized this way and doped with small molecule counter ions is insoluble in any solvent and large size sample preparations are a challenge [32].When polymerization is carried out in the presence of aqueous polyelectrolyte poly(styrenesulfonate) (PSS), it becomes water dispersible which is stable, easy to process, with good film forming properties, and with high visible light transmittance. PSS acts as a template during polymerization and charge balancing counter ion hence keeping the cationic PEDOT segments dispersed in aqueous medium. The molecular weight of PEDOT and PSS is about 1000–2500 g/mol (around 10 to 20 monomer units) and 400,000 g/mol, respectively. PEDOT:PSS in the aqueous media (and the as-prepared film too) has core-shell structure (Figure 2) where the core is conductive PEDOT-rich and the shell is insulator PSS-rich. The hydrophobic PEDOT and hydrophilic PSS nature led to the core shell structure [45]. PEDOT:PSS films prepared from aqueous dispersions have lower conductivity (<1 S/cm) than PEDOT films prepared by oxidative and vapor phase polymerization and stabilized with small molecule counter ions. The main reason for the low conductivity is the core-shell structure which leads to an increase in the energy barrier for charge transport across PEDOT chains by the insulator PSS-rich shell and charge localization due to the coiled PEDOT-rich core [46].

The conductivity can be enhanced up to four orders of magnitude by treatment with polar solvents like dimethyl sulfoxide, ethylene glycol, acids and alcohols called “secondary dopants”. Secondary dopants are different from primary dopants in that they are apparently “inert” and the newly enhanced property persists even upon complete removal of the secondary dopants. Generally, the treatment methods can be grouped into three types: mixing secondary dopant in to the aqueous PEDOT:PSS dispersion, film treatment after drying with secondary dopant or a combination of both methods. The exact mechanism of conductivity enhancement is still a topic of intense investigation. Shi et al. have nicely reviewed treatment methods for conductivity enhancement and mechanism of conductivity enhancement [28]. The additives bring about charge screening between PEDOT and PSS due to their high dielectric constant leading to phase separation. The PEDOT chains will be free to be linearly oriented (from coiled structure), and hence, have a more compact structure (smaller π–π stacking distance), leading to stronger inter chain coupling and better crystallinity with larger crystal size [47]. In the case of post treatment, the excess PSS will also be removed [48]. All these combined effects will lead to increases in carrier concentration and mobility [49,50]. 

There are different grades of PEDOT:PSS commercially available with different conductivities, may be due to the molecular weight difference of PEDOT. Recently, the most extensively used high conductivity grade is Clevios PH1000. Rigorous work has shown very high conductivities of 4700 S/cm for PEDOT:PSS [44] and 7619 S/cm for single crystal PEDOT nanowires [51]. With such improved conductivities, further advancements in different applications are expected.

## 4. PEDOT:PSS Based Conductive Polymer Composites

### 4.1. Conductive Polymer Composites

Metal-based interconnects have been reported to have the highest conductivity, but are not stretchable enough, while elastomeric interconnects are not conductive enough. Conventional conductive polymers such as PPy and PEDOT show promising conductivity for these applications; however, their mechanical properties, biocompatibility and processability still needs improvement [34]. This has led to more attention being directed towards conductive polymeric composites at improved electrical conductivity and mechanical stability. One way to increase the mechanical robustness of conductive polymers is by making a composite with commodity polymers. Composite materials based on conjugated conducting polymers and non-conducting polymers often show a low percolation threshold and improved environmental stability with respect to the conjugated polymer. For instance, compounding techniques used for processing of conventional thermoplastics have been applied to prepare composites of PPy with certain thermoplastics which provided a drastic increase in oxidation stability [53]. In particular, composite of conductive polymer with elastomers have been demonstrated for stretchable/elastic conductive materials/devices. Typical examples of conductive polymer composites for different applications include an electrically conductive PEDOT:PSS-polyurethane (PU) [54]. Table 2 presents non-exhaustive lists of common conductive polymer composites with their suggested application areas.

### 4.2. PEDOT:PSS Based Conductive Polymer Composites

PEDOT:PSS is well known for its high conductivity and applications in conductive textiles and has been used with encouraging results for different applications. Unfortunately, the use of pure PEDOT:PSS is currently constrained by its brittleness. As outlined earlier, one way to improve its mechanical flexibility is to make a composite with traditional commodity polymers. Giuri et al. reported are GGO-PEDOT composites with thermal stability up to 270^°^C for super capacitors [41], Hilal and Han developed a graphene (G) and PEDOT:PSS composites with improved electrical conductivity by 63% of a pristine PEDOT:PSS for solar cells [64]. Taroni et al. reported a thermoelectric PEDOT:PSS/PU blend [38] with improved ductility while maintain reasonable conductivity. A polyvinyl alcohol (PVA) combined with phosphoric acid and PEDOT:PSS and silver flakes that withstands about 230% strain before fracture was reported by Houghton et al. [65]. Furthermore, a PEDOT:PSS-based multi-layer bacterial composite was developed by embedding an electro-active bacterium inside a conductive three-dimensional PEDOT:PSS matrix to increase the electron transfer through the PEDOT:PSS [66]. Table 3 presents non-exhaustive lists of PEDOT:PSS composites with their preparation technique, properties and proposed applications.

## 5. Methods of Treating Textiles with PEDOT:PSS

PEDOT:PSS can be applied on textile materials by carrying out an in-situ polymerization of 3,4-ethylenedioxythiophene (EDOT) on the textile substrate in the presence of PPS or by applying the polymer PEDOT:PSS dispersion onto a textile substrate. In general, adding the polymer into a polymer solution during fiber spinning, coating/dyeing textile substrates (fibers, yarns, fabrics) and/or printing textile fabrics, can be used to produce PEDOT based conductive textiles.

### 5.1. Conductive Fiber Spinning

**Figure 3:**
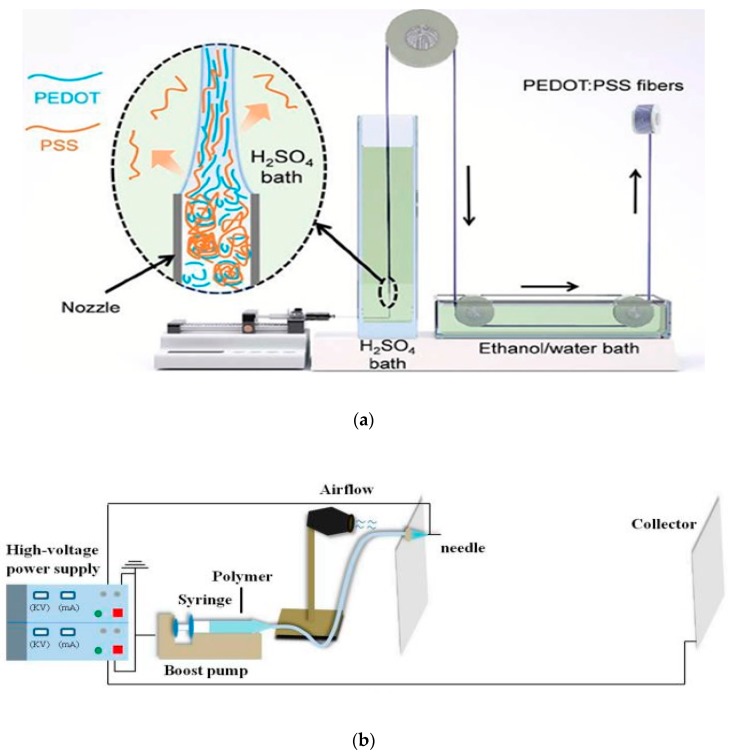
Fiber spinning process: (**a**) schematic illustration modified set-up used in wet-spinning PEDOT:PSS fibers. Inset shows the schematic illustration of the alignment of PEDOT:PSS chains during fiber formation and the outward diffusion of excess PSS to H_2_SO_4_ coagulation bath. Adapted from [74]; (**b**) schematic diagram of electrospinning setup; the distance between the needle and collector was 120 cm. Adapted from [77].

**Figure 4:**
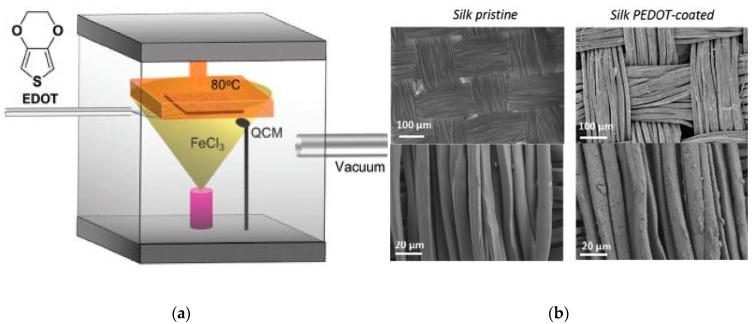
(**a**) The schematic illustration of vapor deposition system for PEDOT; (**b**) SEM images of pristine silk textile and PEDOT-coated silk textile. Adapted from [83].

In this technique, PEDOT:PSS is added to a conventional polymer solution during fiber wet spinning or electrospinning (Figure 3a) in order to produce a conductive fiber or filament, or the PEDOT:PSS alone can be spun in to a fiber. In 2003, Okuzaki and Ishihara presented their first study on the manufacture of 4.6 to 16 μm PEDOT:PSS microfibers using wet-spinning technique with an electrical conductivity of 0.1 S/cm [7]. The Young’s modulus, tensile strength, and elongation at break for the resulting microfibers were 1.1 GPa, 17.2 MPa, and 4.3%, respectively. Jalili et al. reported a simplified wet-spinning process for continuous PEDOT:PSS fibers which showed a conductivity up to 223 S/cm by post treatment of the fibers with ethylene glycol [71]. In another approach, they used an aqueous blend of PEDOT:PSS and poly(ethlylene glycol) and the conductivity of the fibers increased by a 30-fold (from 9 to 264 S/cm) without the need of a post treatment. Okuzaki et al. developed PEDOT:PSS microfibers with diameter of ca. 5 µm by wet-spinning [72]. They improved the electrical conductivity of the fibers from 74 S/cm to 467 S/cm by subsequent dip-treatment of the fibers in ethylene glycol. The mechanical properties of the microfibers were also improved by the dip-treatment; the Young’s modulus and tensile strength increased from 3.2 GPa and 94 MPa to 4.0 GPa and 130 MPa, respectively. Zhou et al. further enhanced the electrical conductivity of wet spun PEDOT:PSS microfibers to 2804 S/cm via wet-spinning followed by post treatment with ethylene glycol and hot-drawing [73]. This high conductivity is due to the combined effects of the vertical hot-drawing process and doping/de-doping of the microfibers with ethylene glycol. Moreover, they had a semiconductor metal transition at 313 K with superior mechanical properties with a Young’s modulus up to 8.3 GPa, a tensile strength reaching of 409.8 MPa and a large elongation before failure (21%). J. Zhang et al. also carried out a wet spinning of PEDOT:PSS fiber and obtained better conductivity of PEDOT:PSS fiber, 3828 S/cm, by decreasing the fiber diameter using a fine gauge needle [74]. The wet-spinning set-up was modified as shown in Figure 4a. Liu et al. also reported composite conductive fibers based on PEDOT:PSS blended with polyacrylonitrile [75] by wet spinning. Fibers with 1.83 wt% of PEDOT:PSS showed a conductivity of 5.0 S/cm. Seyedin et al. demonstrated a scaled-up fiber wet-spinning production of electrically conductive and highly stretchable PU/PEDOT:PSS fibers which were then used in knitting for a knee sleeve prototype with application in personal training and rehabilitation following injury [3]. The fiber showed a conductivity of 166 S/cm very close to pristine PEDOT:PSS film with a wide strain sensing capability up to a 260 % strain. 

Jin et al. employed an electrospinning and in-situ synthesis process to fabricate silver nanoparticles coated PEDOT:PSS/PVA flexible self-supporting nanofibers with greatly improved electrical conductivity [76]. Q. Zhang et al. also used an electrospinning to fabricate a PVA/PEDOT:PSS nanofiber with an average diameter of 68 nm for a gas sensor (Figure 3b) [77].

### 5.2. Polymerization of PEDOT on the Textile Substrate

The PEDOT monomer can be polymerized on the textile substrate (fiber, yarn, fabric or garment form) by in situ, vapor phase or electrochemical polymerization by using EDOT and appropriate chemicals like oxidants [78]. This method combines polymerization of the PEDOT and coating of the textile.

The attachment of PEDOT on the fabric surface depends on the chemistry of the fiber as well as the surface roughness of the fiber. Though direct polymerization of PEDOT on the textile seems straight forward, it is difficult to control the parameters. Moreover, it is used for small sample size and a challenge for industrial requirements. Hong et al. carried out five cycles of in-situ polymerization of PEDOT on poly(trimethylene terephthalate) fabrics in the presence of ferric p-toluenesulfonic acid and ferric chloride as oxidants followed by butane treatment and obtained an electrical conductivity of 3.6 S/cm [79]. Bashir et al. reported an electrically conductive polyester fabric with an electrical resistance of ~2000 Ω, coated by PEDOT through oxidative vapor phase polymerization (VPP) in the presence of Fe (III) chloride hexahydrate oxidant [80]. They also obtained electro-conductive aramid, viscose and nylon fabrics by the same approach. In another work, they produced a conductive viscose yarn with electrical resistance 6 kΩ by oxidative chemical vapor deposition, by removing the impurities like acetone and ethyl acetate, prior to the oxidant enrichment and polymerization steps [81]. Trindade et al. also coated a polyester fabric by PEDOT through VPP and obtained a lower sheet resistance of~20 Ω/sq by increasing the concentration of the oxidant, Fe (III) chloride hexahydrate [82]. L. Zhang et al. coated textile fabrics (silk, linen, wool, pineapple, bamboo rayon) by PEDOT through VPP and obtained a sheet resistance from 200 to 9.46 kΩ depending on the porosity of the fabric; porous fabric gives higher sheet resistance than tight-fabric [83]. Overall, the electrical and mechanical properties of conductive textiles are determined by the concentration of oxidants, pretreatment of the original pristine fabric and post-treatments of the conductive fabric, type and form of textile substrate and the polymerization conditions. The illustration of vapor deposition system is shown in Figure 4.

### 5.3. Coating/Dyeing of Textiles with PEDOT:PSS

**Figure 5:**
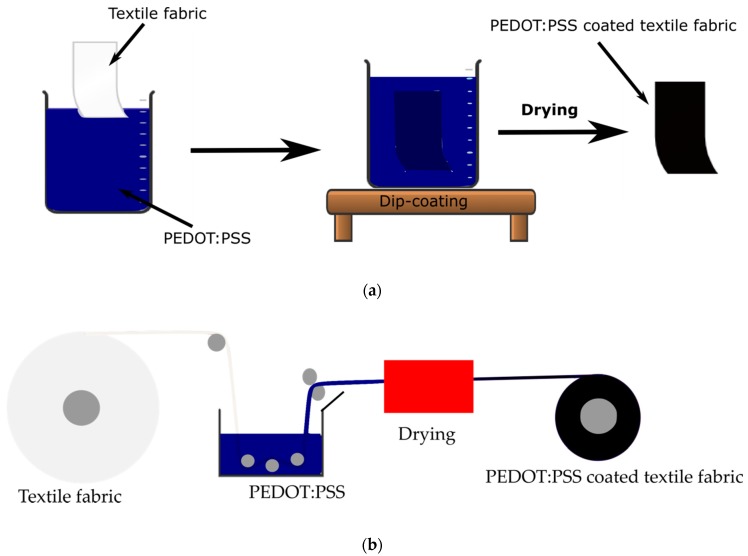
Schematic representation of the setup for dyeing: (**a**) discontinuous process; (**b**) continuous process.

In the coating/dyeing method, the appropriate form of textile is treated by immersing/dipping in PEDOT:PSS dispersion with appropriate auxiliary chemicals. This is method mimics either the exhaust or continuous dyeing method of commercial textile processing. It is the most popular method practiced for making conductive textiles with PEDOT:PSS. The uniformity as well depth of dyeing/coating depends on the functional group of the textile. Ding et al. treated cotton, cotton/polyester, polyester and nylon/spandex fabrics by impregnating with PEDOT:PSS and showed that conductivity is higher for fabrics which swell well in water [84]. Ryan et al. dyed up to 40 m long silk yarn with PEDOT:PSS with conductivity of ~14 S/cm which was durable to machine washing [85]. The reason to wash durability of PEDOT:PSS on silk is due to the dyeing effect and the presence of a fluorosurfactant Zonyl FS-300 used during dyeing. When cotton was dyed by the same method, it was too fragile due to hydrolysis of the cellulose by the strong acidic PEDOT:PSS. The same group further demonstrated a continuous dyeing process to produce more than 100 m of silk thread dyed with PEDOT:PSS for a wash and wear resistant functional thread with a conductivity of about 70 S/cm [86]. Ding et al. produced PU fibrous nonwoven and treated it with PEDOT:PSS by dip-coating [2]. The PEDOT:PSS@PU nonwovens showed sheet resistance of 35–240 Ω/sq (electrical conductivity of 30–200 S/m) by varying the number of dip-coating times. This conductive nonwoven maintained its surface resistance up to 50% strain, promising for wearable application. Tadesse et al. also treated polyamide/lycra elastic fabric with PEDOT:PSS by dipping only once and showed a sheet resistance of ~ 1.7 Ω/sq [87]. The fabric was stretchable up to ~650% and maintained reasonable conductivity up to washing cycles. The durability to washing in this case is also due to dyeing effect where there is some kind of chemical interaction between the fiber and PEDOT:PSS. A schematic representation of discontinuous and continuous PEDOT:PSS dip-coating/dyeing on a textile fabric are shown in Figure 5.

### 5.4. Printing of PEDOT:PSS on Textile

**Figure 6:**
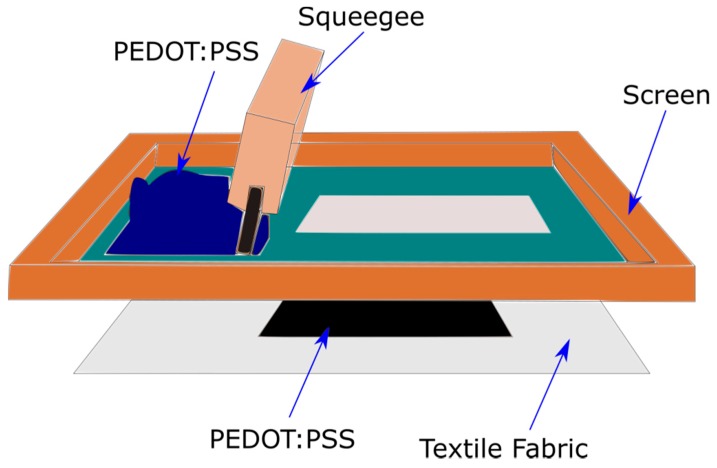
Schematic illustration of PEDOT:PSS screen printing on a textile fabric. Adopted from [91].

Printing is a well-developed textile processing method used industrially is also used to apply the PEDOT:PSS to the textile structure in the presence of thickening agents to obtain an adequate paste or ink viscosity. Guo et al. demonstrated a fabrication of all-organic conductive wires by utilizing patterning techniques such as inkjet printing and sponge stencil to apply PEDOT:PSS onto a nonwoven polyethylene terephthalate (PET) providing a wide range of resistance, i.e., tens of kΩ/sq to less than 2 Ω/sq that allows the resistance to be tailored to a specific application [88]. Sinha et al. demonstrated the integration of screen-printed PEDOT:PSS electrocardiography (ECG) circuitry on finished textiles and recorded an ECG signal comparable to Ag/AgCl connected to copper wires [89]. Zhao et al. also used screen-printing to produce a PEDOT:PSS and carbon-based disposable electrochemical sensor for sensitive and selective determination of carmine [90]. Tseghai et al. used a flat screen printing to coat a PEDOT:PSS conductive polymer composite on to a knitted cotton fabric and obtained a sheet resistance of 24.8 kΩ/sq [91]. The conductive textile fabric stays conductive until its infliction point of stretching. The schematic illustration of screen printing is shown in Figure 6.

## 6. Applications of PEDOT:PSS-based Conductive Textiles

As outlined earlier, PEDOT:PSS has high electrical conductivity, thermal stability, decent biocompatibility, and is solution processable. These interesting properties make it attractive for different textile-based applications including sensors, energy harvesting, and storage devices.

### 6.1. Sensors

**Figure 7:**
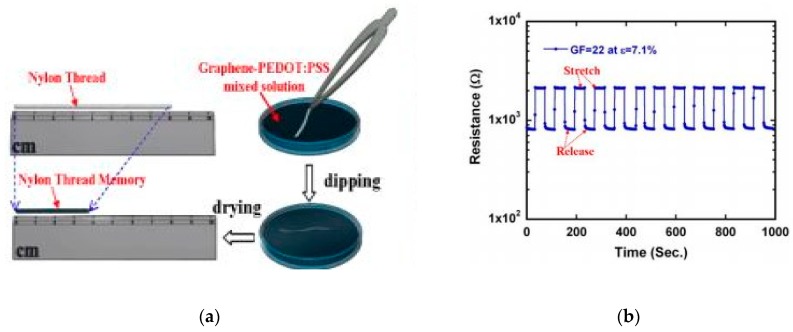
A graphene-PEDOT:PSS coated nylon thread: (a) schematic of the simple two-step dip-and-dry solution process for the fabrication (right) and the actual picture of the sample with the length reduction from 80 to 29.48 mm after dip-and-dry; (**b**) resistive memory strain sensor thread at many stretch and release cycle under applied a fixed ε = 7.1%. Adopted from [92]

The demand of textile-based sensors is increasing because of their lightweight, flexiblity, and possibility of washing. PEDOT:PSS-based textiles have been widely used as a sensing component for strain, pH, humidity, biopotential, and temperature. Zahid et al. applied graphene nanoplatelets dispersed in PEDOT:PSS solutions for producing a conductive, breathable and lightweight mercerized cotton fabrics by spray coating which showed a highly repeatable and stable response to cyclic deformation tests at 5% and 10% strain rates for up to 1000 cycles with ~90% viscoelastic recovery levels after cessation [68]. Kang reported a resistive memory graphane-PEDOT:PSS coated nylon thread with a strain response for wearable applications as an example of bio-potential sensors (Figure 7) [92]. Seyedin et al. developed a strain sensor from a PU/PEDOT:PSS fibers with conductivity of 9.4 S/cm [3]. The resistance of this textile sensor stays stable up to 160% strain and up to 500 cycles. The high conductive textile-based hybrid showed high stability during stretching. Pani et al. developed a new textile ECG electrode based on woven fabrics treated with PEDOT:PSS for bio-potential recordings tested on human, both in terms of skin contact impedance and quality of ECG signals recorded at rest and during physical activity [93]. The electrode was found to be capable of operating under both wet and dry conditions, which could be an important milestone in wearable monitoring of heart. Ankhili et al. developed an ECG sensor electrode from a PEDOT:PSS screen-printed cotton fabric and obtained a clear ECG wave amplitudes up to 50 washing cycles [94]. The same group also produced washable screen-printed cotton textile electrodes with and without lycra of different PEDOT:PSS concentration, providing a medical quality ECG signal to be used for long-term ECG measurements with a similar result to silver-plated cotton fabric at 12.8 wt% of PEDOT:PSS to pure cotton [95]. Niijima et al. produced "hitoeCap" from PEDOT:PSS textile electrodes for securing electromyography of the masticating muscles [96].

Furthermore, Abbasi et al. worked on the use of PEDOT:PSS material for the implementation of high sensitivity moisture sensor devices, which showed significant frequency shifts [97]. They demonstrated sensing capacity even for small moisture variations. Smith et al. developed a wearable pH sensor cotton yarn in PEDOT:PSS and multi-walled carbon nanotubes followed by PANI deposition that produced electrodes with significant biocompatibility and antibacterial properties that could be manufactured (alongside quasi-reference electrodes) into wearable solid-state pH sensors and achieved wearable pH sensors [98].

### 6.2. Energy Harvesting and Storage

**Figure 8:**
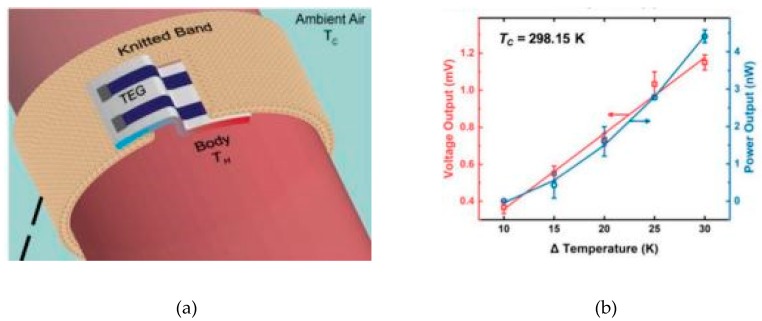
(**a**) Design schematic of a wearable thermoelectric generator; (**b**) thermoelectric power and voltage outputs for a tobacco cotton thermopile at 25 °C. Adapted from [101].

Textiles coated with PEDOT:PSS have been used to manufacture flexible and lightweight energy harvesting and storage devices. This is quite interesting to power from wearable electronics to medical implantable devices. PEDOT:PSS is a promising organic thermoelectric material, materials which change temperature difference into electricity or vice versa [52]. PEDOT:PSS coated textiles have been studied for wearable thermoelectric applications to harvest the temperature difference between the body and outer surrounding. Du et al. coated polyester fabric strips with PEDOT:PSS where the flexibility and air permeability was not affected and attached them on non-coated [99]. The treated fabric showed electrical conductivity of ~1.5 S/cm and generated thermoelectric voltage of 4.3 mV at a temperature difference of 75.2 °K. Ryan et al. dyed silk yarn with PEDOT:PSS and made 26 thermoelectric legs by embroidering on felted wool fabric which showed a thermoelectric voltage output of ~351 μV/K [85]. These PEDOT:PSS dyed silk yarns were stable to machine washing and up to thousand bending cycles. Recently, Jia et al. coated textile with PEDOT via VPP which combined thermoelectric generation and strain sensing application [100]. Allison et al. used vapor printing method on commercial cotton fabric to make all textile wearable band which generated thermovoltages as high as 20 mV when worn on the hand (Figure 8) [101].

Supercapacitors are alternative energy storage devices for fast charge discharge applications. Textile based supercapacitors have attracted attention due to their inherent flexibility and their potential use in wearable electronics. Nuramdhani et al. demonstrated that PEDOT:PSS sandwiched between two stainless steel conductive yarns showed capacitive behavior as an energy storage device [26]. Ma et al. reported flexible stainless steel/cotton blend yarn coated with PEDOT:PSS and PPy which can be cycles up to 5000 cycles [102]. Yuan et al. reported fiber shaped yarn supercapacitors by twisting wet spun PEDOT:PSS which showed a high areal capacitance of 119 mF/cm^2^ [103]. Li et al. prepared flexible textile supercapacitors by spray coating of graphene nanosheets and PEDOT:PSS which exhibit an enhanced specific areal capacitance of 245.5 mF/cm^2^ [104]. Yuksel et al. reported cotton fabric coated with MnO_2_/SWNT/PEDOT:PSS ternary nanocomposite supercapacitors that gives a specific capacitance up to 246 F/g and areal capacitance of 64.5 mF/cm^2^ [105]. 

### 6.3. Other Applications

There is a strong need in flexible and wearable actuators, organic light-emitting diode (OLED) and antenna. The inherent properties of PEDOT:PSS make it ideal to fabricate these devices on textiles. For instance, Li et al. developed a screen-printed textile patch antenna capable of operating at 2.4 GHz by using PEDOT:PSS as a patch and ground on polyester fabric [106]. The antenna is flexible and breathable which make it well-fit for wearable applications. 

**Figure 9:**
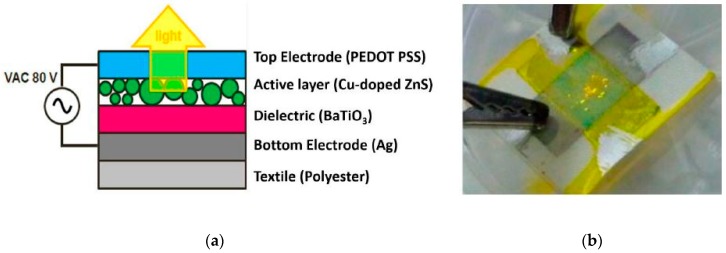
(**a**) Build-up of the alternating current powder electroluminescence technology; (**b**) OLED stack printed on a textile. Adapted from [108].

Actuation is another application area of smart textiles. Miura et al. developed a foldable PEDOT:PSS/PVA fiber by wet spinning that exhibits a repeatable contraction motion at air by applying alternating square-wave voltages between 0 and 8 V [107]. Verboven et al. reported an OLED with maintained textile properties by screen printing of silver as a bottom electrode, barium titanate as a dielectric, copper-dopped zinc-oxide as an active layer and PEDOT:PSS as a top electrode on polyester fabric that requires 3–5 V power supply [108]. The thickness of the OLED on the textile fabric was only 0.5 µm which is a good platform for wearable application; the schematic illustration and actual OLED are shown in Figure 9.

## 7. Conclusions and Outlook

Current advances in textile technology, new materials, nanotechnology, and miniaturized electronics make wearable systems more feasible, but fit comfort is the ultimate key factor for wearable device user acceptance. It is convincing that this objective can only be achieved by addressing mechanical robustness and material durability in what is recognized as a harsh electronic environment: the human body and society. Thus, the use of conductive polymer composites for smart textiles could possibly be the primal solution. Composites of conductive polymers have been explored to overcome their brittleness and processability, while retaining their electrical conductivity and desirable biological properties such as cell adhesion. Enhanced mechanical properties of conductive composites usually come at the expense of desirable electrical conductivity of conductive polymers. On the other hand, fundamental understanding of the interaction between the conductive polymer filler and the non-conductive commodity polymer matrix will lead to get synergistic effect in mechanical performance and electrical properties of the composites. There is a need to achieve reasonable electrical conductivity with the lowest possible amount of conductive filler, while retaining the properties of the host polymer. The major challenges thus lie in selection of conductive filler achieving low percolation threshold and retaining biocompatibility for biomedical applications. PEDOT:PSS-based conductive polymer composites are promising for the manufacturing of smart textiles with better biocompatibility, flexibility, conductivity, printability, miscibility and weight, and as such much better suited for wearable applications compared to the common electrodes such as metallic coatings and others. As a result, tremendous PEDOT:PSS-based conductive textiles have been developed by different approaches as sensor, energy harvesting devices, antennas, OLEDs etc. However, the conductivity stability of PEDOT:PSS conductive polymer composites after being applied on textile substrates still needs an improvement. This improvement could be on the synthesis of PEDOT:PSS itself, on the combination and proportion of the polymers in the composite or by seeking new approaches of integration.

## Figures and Tables

**Table 1:** Non-exhaustive conductivity and properties of common conductive polymers.

Polymer	Conductivity (S/cm)	Doping	Properties	Limitations	Ref.
PPy	2000	P	High electrical conductivity, ease of preparation and ease of surface modification	Rigid, brittle and insoluble	[42]
PANI	112	P	Diverse structural forms, environmentally stable, low cost	Hard to process, non-biodegradable, limited solubility	[43]
PTh	560	P	High electrical conductivity, ease of preparation, good optical property	Hard to process	[29]
PEDOT:PSS	4700	P	High electrical conductivity used as transparent electrode	Needs additional steps to process	[44]

**Table 2:** Properties of common conductive polymer composites.

Conductive Polymer Composite	Conductivity (S/cm)	Properties	Suggested Application	Ref.
PPy/Hyaluronic Acid	3.1 × 10^−3^	Can support tissue growth and stimulate specific cell functions	Tissue engineering and wound-healing	[55]
PANI Nanofibers/Collagen	0.27	Well suited for cell culture	Scaffold Materials for biomedical	[56]
PPy/Chitosan	10^−3^–10^−7^	Radical scavenger	Food packaging and biomedical	[57]
PEDOT:Tos/Glycol	1486	Soft, flexible and biocompatible	Implantable devices	[58]
PPy/Cellulose Acetate	6.9 × 10^−4^–360	Soft and flexible and	Wearable electronics	[59]
PANI Nanoparticles/Polyacrylic Acid/Polyvinyl Alcohol	0.04–0.06	Hydrogel, biocompatible, good mechanical strength and good swelling properties	Strain sensor	[60]
Polythiophene derivative/PU	2.2 × 10^−5^	Suitable for supporting electrically stimulated cell growth	Tissue engineering	[23]
PEDOT:PSS/PU/Ionic liquid	8.8 × 10^−5^	Mechanically flexible and stretchable	Actuating devices	[61]
PPy/poly(D,L-Lactic Acid)	5.7 × 10^−3^–15.7 × 10^−3^	Nerve tissue regeneration, biocompatibility	Synthetic nerve conduits	[62]
PPy nanoparticles/PU	2.3 × 10^−6^	Cytocompatible, elastomeric properties	Tissue engineering	[63]

**Table 3:** Properties of common PEDOT:PSS based conductive polymer composites.

Conductive Polymer Composite	Resistivity, Ω cm (Resistance, Ω/sq)	Properties	Manufacturing Technique	Proposed Application	Ref.
GO/rGO filled PVA/PEDOT: PSS	10^7^	Highly flexible free-standing	Solvent casting	Strain sensor	[67]
PEDOT:PSS/PU	(35−240)	Highly flexible, stretchable	Electrospinning	Strain sensor	[2]
PEDOT:PSS/Bacteria	103^a^	20 times more steady-state current than native biofilms baseline with signal level of 6.31 μA/cm^3^	Embedding bacteria into electro-polymerized PEDOT:PSS on carbon felt anodes	Bioelectronics	[66]
PEDOT:PSS/PU	1.26 × 10^−2^	Sensitivity to different stimuli including strain, ambient temperature and/or air flow high electrical conductivity	Dispersion mixing	Stretchable self-powered sensors	[38]
PEDOT:PSS/graphene	(25)	Strong resistance against fatigue upon repeated folding-unfolding	Spray coating	Data storage and transmission, biosensors and actuators	[68]
3D Graphene/PEDOT:PSS	4.1 × 10^−2^	Good resistance retention capability under deformations	Graphene networks coated by PEDOT:PSS	Next-generation stretchable electronics	[69]
CNT/PEDOT:PSS	3 × 10^−3^	Good thermoelectric performance	Vacuum assisted filtration method and H_2_SO_4_ treatment	Flexible thermoelectric generator	[70]

^a^ The unit for the value of Ref. [66] is Ω.
